# The Autoimmune Skin Disease Bullous Pemphigoid: The Role of Mast Cells in Autoantibody-Induced Tissue Injury

**DOI:** 10.3389/fimmu.2018.00407

**Published:** 2018-03-01

**Authors:** Hui Fang, Yang Zhang, Ning Li, Gang Wang, Zhi Liu

**Affiliations:** ^1^Department of Dermatology, Xijing Hospital, Fourth Military Medical University, Xi’an, China; ^2^Department of Dermatology, School of Medicine, University of North Carolina at Chapel Hill, Chapel Hill, NC, United States; ^3^Department of Dermatology, The Second Hospital, School of Medicine, Xi’an Jiaotong University, Xi’an, China; ^4^Department of Microbiology and Immunology, School of Medicine, University of North Carolina at Chapel Hill, Chapel Hill, NC, United States; ^5^Lineberger Comprehensive Cancer Center, University of North Carolina at Chapel Hill, Chapel Hill, NC, United States

**Keywords:** autoantibodies, bullous pemphigoid, hemidesmosome, mast cells, skin autoimmunity

## Abstract

Bullous pemphigoid (BP) is an autoimmune and inflammatory skin disease associated with subepidermal blistering and autoantibodies directed against the hemidesmosomal components BP180 and BP230. Animal models of BP were developed by passively transferring anti-BP180 IgG into mice, which recapitulates the key features of human BP. By using these *in vivo* model systems, key cellular and molecular events leading to the BP disease phenotype are identified, including binding of pathogenic IgG to its target, complement activation of the classical pathway, mast cell degranulation, and infiltration and activation of neutrophils. Proteinases released by infiltrating neutrophils cleave BP180 and other hemidesmosome-associated proteins, causing DEJ separation. Mast cells and mast cell-derived mediators including inflammatory cytokines and proteases are increased in lesional skin and blister fluids of BP. BP animal model evidence also implicates mast cells in the pathogenesis of BP. However, recent studies questioned the pathogenic role of mast cells in autoimmune diseases such as multiple sclerosis, rheumatoid arthritis, and epidermolysis bullosa acquisita. This review highlights the current knowledge on BP pathophysiology with a focus on a potential role for mast cells in BP and mast cell-related critical issues needing to be addressed in the future.

## Mast Cells (MCs) and MC Receptors

Mast cells are derived from hematopoietic progenitor cells and have been considered as a central player in functional interaction between innate and adaptive immunity. MCs are initially located in the blood vessel and the lymphatic system before homing to tissues, where they acquire their final effector characteristics (1). There are at least two subpopulations of murine MCs based on the composition of chymases and tryptases within their granules. While MC_T cell_s are the prominent MC type within the mucosa of the respiratory and gastrointestinal tracts, MC_TC_ cells are localized within connective tissues including the dermis, submucosa of the conjunctivae, gastrointestinal tract, heart, and perivascular tissues (2). The maturation of MCs in the tissue mainly relies on stem cell factor (SCF) expressed on the homing tissue, which is the ligand of KIT (1).

Mast cells express KIT (CD117) and FcεRI on their surface, which are the receptors of SCF and IgE, respectively. MCs also express other cell surface receptors, including IgG receptors (FcγRIII, FcγRIIa, and FcγRI), C3a and C5a receptors (C5aRs), Toll-like receptors, and receptors for many cytokines/chemokines (3). These receptors mediate activation of MCs. Upon activation, MCs release their mediators to the homing sites, which act in host defense and various pathological conditions (4). Mediators produced by MCs are divided into two categories: preformed and newly synthesized (5). Many mediators are preformed and stored in granules, such as histamine, serine proteases (tryptase and chymase), and TNF-α (6). Upon activation of MCs, these preformed mediators are released into the extracellular environment within minutes (7–9).

After the initial activation, the synthesized bioactive metabolites of arachidonic acid, prostaglandins, leukotrienes (LTs), and cytokines/chemokines will be released into the affected tissue sites rapidly. The second release of granules will amplify the immediate hypersensitivity reaction through the interaction with local cells and infiltrating immune cells (4).

## MCs in Non-Skin Autoimmune Diseases Multiple Sclerosis (MS) and Rheumatoid Arthritis

Mast cells have been considered as key effector cells in many immune activities, especially IgE-associated immune responses, including host defense to parasites, allergic diseases, chronic inflammatory disorders (10, 11), and cancer (12, 13). MCs have also been implicated in autoimmune diseases (14–19), such as MS, rheumatoid arthritis (RA), and the autoimmune skin blistering diseases bullous pemphigoid (BP) and epidermolysis bullosa acquisita (EBA).

Multiple sclerosis is an autoimmune disease of the central nervous system characterized by chronic inflammation and progressive demyelination (20). MCs and activated MCs are present in the target tissues of MS patients and correlated with disease severity (21–24). The animal model of MS, experimental autoimmune encephalomyelitis (EAE), can be induced by active immunization of susceptible mouse strains with myelin components such as myelin basic protein and myelin oligodendrocyte glycoprotein (MOG) (25). RA is an autoimmune disease of the joints characterized by chronic inflammation and cartilage destruction (26). Increased MCs and MC-derived inflammatory mediators are found in the inflamed joints of RA patients (27–29). K/BxN mouse serum contains autoantibodies against the glucose-6-phosphate isomerase and, when passively transferred to mice, induces experimental RA (30).

## Role of MCs in Experimental MS and Rheumatoid Arthritis

Mast cell-deficient mice have been widely used to determine the role of MCs in various physiological and pathological conditions, including autoimmune diseases. Whether MCs actively participate in the pathogenesis of MS and RA has been extensively debated recently due to controversial results obtained from different MC-deficient mouse strains. For a more comprehensive and in-depth review, please refer to the studies by Yu et al. and Rivellese et al. (15, 31). In MOG-induced EAE, MC-deficient Kit*^W/W-v^* mice (caused by Kit mutations) developed a significantly reduced disease, and reconstitution of MC-deficient Kit*^W/W-v^* mice with wild-type bone marrow-derived MCs restored the disease (32). Similarly, MC-deficient Kit*^W/W-v^* mice were protected from K/BxN serum-induced RA (33). K/BxN serum also failed to induce RA in Mgf*^Sl/Sl-d^* mice, another MC-deficient strain caused by mutations in the gene encoding the Kit ligand SCF (33). Since MC deficiency by Kit or SCF mutations also caused a variety of immunological abnormalities, new Kit-independent MC-specific deletion mouse strains were developed recently. It turned out that MCs were not required in the development of EAE and serum-induced RA (34).

## BP: Clinical and Immunohistological Features

Bullous pemphigoid is an autoimmune subepidermal blistering disease induced by autoantibodies against the two components of the hemidesmosome, BP180 and BP230. BP is the most common autoimmune blistering disease and most prevalent in the elderly. BP typically presents with tense, mostly clear blisters, and erythema, frequently in conjunction with urticarial plaques (35). Blisters occur on either a normal or a erythematous base, containing serous or serosanguinous fluid (36). The disease has a symmetric distribution, and the predilection sites include the lower abdomen, flexor surfaces of the limbs, groin, and axillae (37). In almost all patients, severe pruritus is present. About 10–20% of patients show mucosal involvement, with the oral mucosa being the most common mucosal site (38, 39). Two prospective studies showed that up to 20% of patients with BP have no obvious blistering at the time of diagnosis (38–40).

**Figure 1:**
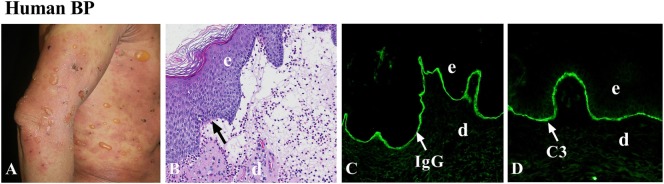
Human bullous pemphigoid (BP). **(A)** Large, tense bullae, and erythematous patches seen in BP patient. **(B)** Histology reveals dermal–epidermal junction separation with inflammatory cell infiltration. Immunofluorescence shows linear deposition of IgG **(C)** and complement C3 **(D)** at the basement membrane zone (BMZ). d, dermis; e, epidermis. Arrow, the BMZ. Original magnification, 100× for panels **(B–D)**.

Histopathologically, hematoxylin and eosin staining of early bulla in BP reveals subepidermal blistering with dense inflammatory infiltrate consisting predominantly of eosinophils, but also lymphocytes, neutrophils, and MCs. Eosinophils are seen within the blister and in the edematous papillary dermis (41). In the early non-bullous phase, subepidermal clefts and eosinophilic spongiosis (epidermal spongiosis with eosinophils within the epidermis) can be found (41). Therefore, BP is an autoimmune and inflammatory disease (Figure 1). Direct immunofluorescence staining exhibits linear deposition of IgG and/or complement components (C3 and/or C5) at the dermal–epidermal junction. IgG deposition sometimes is combined with weaker linear IgA or IgE staining. To identify circulating autoantibodies to the DEJ, indirect immunofluorescence (IIF) with normal human skin as the substrate is usually examined. Artificial blisters can be induced by incubating the skin specimen with 1 M NaCl solution. Since BP180 and BP230 are on the epidermal side of the artificial blisters, autoantibodies from BP patients are known to react with the epidermal side of the blisters (42). In contrast, autoantibodies from other autoimmune blistering diseases, including EBA and anti-laminin γ1 pemphigoid, react with the dermal side of the artificial blisters (35). Thus, IIF with the salt-split skin as a substrate is helpful in distinguishing BP from other autoimmune blistering disorders.

## BP Autoantigens

Bullous pemphigoid autoantibodies target two hemidesmosomal components BP180 (BPAG2) and BP230 (BPAG1), which are involved in dermal–epidermal cohesion (43–45). BP180 is a type II transmembrane glycoprotein with a globular cytoplasmic domain and a large extracellular region containing 15 collagenous and 16 non-collagenous (NC1-16) domains. The 16th non-collagenous (NC16A) domain is the immunodominant region in BP (46). Anti-NC16A IgG autoantibodies are detected in more than 90% of BP patients (47) and have been shown to be pathogenic in skin organ culture system and in animal models of BP (48–50) (see below). BP230 is a 230-kDa intracellular component of the hemidesmosomal plaque and belongs to the plakin family of proteins. Anti-BP230 autoantibodies are detected in nearly 60% of BP patients (51). In addition to IgG reactivity, anti-BP180/BP230 IgE autoantibodies are present in serum samples from most patients (47, 52, 53).

## Genetics of BP

Genetic, environmental, and stochastic factors contribute to susceptibility to most autoimmune diseases. The human MHC encodes many glycoproteins that include the HLA class I and class II molecules, which provide a pivotal role in the recognition of antigenic peptides by T cells. A lot of polymorphisms of HLA-II class alleles have been identified in several populations of patients with BP (54–58). These polymorphisms HLA class II alleles occur likely due to changes in the charge of the active binding site on the HLA molecules for binding of autoantigenic peptides. A common HLA class II allele, HLA-DQB1*03:01, is positively associated with BP in multiple populations (54, 55, 58) and also appears to be associated with distinct clinical pemphigoid variants (59–61). In addition, the activation of BP180-autoreactive T cells from a cohort of BP patients with HLA-DQB1*03:01 was found to be restricted by this BP-associated HLA class II allele (55).

## T Cell Response in BP

CD4+ T helper (Th) cells are thought to participate in early disease development and perpetuation of autoantibody-mediated autoimmune blistering diseases. Th cells, upon proper costimulation, are activated and produce and secrete distinct cytokines that stimulate B cells. This Th–B cell interaction thus fosters plasma cell development and autoantibody production (62). In BP, autoreactive CD4+ T lymphocytes recognize unique epitopes within the extracellular region of BP180 (63). The majority of BP patients examined have both Th1 and Th2 responses against the BP180 ectodomain (55, 64). BP180-reactive Th cells and IgG autoantibodies recognized similar or identical epitopes clustered in distinct regions of the BP180 ectodomain and BP230 (49, 62, 65). Li et al. found that follicular T helper (Tfh) cells and IL-21 were crucial for the secretion of antibodies against BP180NC16A domain in T cell/B cell co-culture system, indicating that these Tfh cells may be involved in the pathogenesis of BP (66).

## MCs in Human BP

In 1978, Wintroub et al. found that increased MCs and increased degranulation of MCs at the BP lesional sites are the earliest events in BP lesion formation (67). The evolution of clinical BP lesions is associated with a sequence of histopathologic events, starting with MC alternation and proceeding to immune cell infiltration first with lymphocytes followed by eosinophils and basophils. Electron and light microscopy revealed that MCs are mainly present in the papillary dermis adjacent to the dermal–epidermal junction and demonstrate a unique, focal, irregular loss of granule contents (68).

Various inflammatory mediators have been found in lesional/perilesional skin, blister fluids, and/or blood of patients with BP, including C5a, histamine, LTs, and many cytokines/chemokines (e.g., IL-1, IL-2, IL-5, IL-6, IL-8, TNF-α, eotaxins, and IFN-γ) (69–75). These mediators can recruit and directly activate MCs and leukocytes. Moreover, MCs can influence biological responses through the production of multifunctional cytokines and enzymes (76–78). Evidence suggests that metalloproteinase (MMP9 in particular), leukotrienes (LT), heparin and platelet activating factor (PAF) derived from MCs also play a role in the inflammatory process during blister formation (67). Tryptase is a specific proteolytic enzyme synthesized and stored in MCs and released by MCs when activated by various stimulating factors. Tryptase, therefore, is considered a reliable marker for the presence of MCs (79). A previous study showed that tryptase levels in BP blister fluid were increased compared with the respective sera and significantly correlated with several cytokines/chemokines (IL-3, IL-4, IL-5, IL-6, IL-7, IL-8, and RANTES), VEGF, and sICAM-1. Most importantly, the blister fluid tryptase levels were also positively correlated with titers of autoantibodies against basement membrane zone antigens (80), which relates to the severity of the disease. Increased levels of cytokines (including IL-1β, IL-5, IL-6, IL-10, IL-15, and TNF-α) and chemokines (such as CCL2, CCL5, CCL11, CCL13, and CCL18, and IL-8) were identified in serum samples and blister fluids of patients with BP, and some of these mediators parallel disease activity (81). Bieber et al. investigated serum parameters related to activation of different inflammatory cells and found higher serum concentrations of MCs tryptase during ongoing disease. The serum levels of MCs tryptase significantly decreased at the time of clinical remission of the patients. In addition, serum concentrations of MCs tryptase were significantly associated with levels of circulating anti-BP180 autoantibodies (82). These data suggested that increased concentrations of MCs tryptase in BP blister fluids and/or serum partly correlate with cytokines, autoantibodies, and clinical disease severity in BP patients.

BP180-specific IgG autoantibodies are the most abundant immunoglobulin isotype; however, IgE autoantibodies with the same or similar epitope specificity are also present in about 70–90% of BP patients (83, 84). It has been speculated that IgE autoantibody–mediated activation of MCs in the skin may be involved in the development of certain clinical symptoms typical of BP, such as urticarial plaques, dermal edema, and eosinophilic inflammation. Dimson et al. found IgE-coated MCs in the perilesional skin of the BP patients, and BP180 peptides were co-localized on these MCs, suggesting that BP180-specific IgE that bind to the surface of MCs through IgE receptors, when interacting with BP180 peptides, result in MC degranulation. Moreover, basophils obtained from untreated BP patients stimulated with recombinant BP180NC16A released significantly higher histamine compared to NC16-stimulated basophils from normal control or from treated BP patients (83). In addition, Freire et al. reported that IgE co-localized with MCs in the perilesional skin of BP patients, and IgE-BP180 complexes could activate MCs *via* the high-affinity IgE receptor (FcεRI), conceivably triggering MC degranulation-mediated events resulting in tissue inflammation (85).

Omalizumab is a recombinant humanized monoclonal antibody that inhibits the binding of IgE to FcεRI on the surface of MCs and basophils. Patients with BP treated with omalizumab showed reduced disease severity including decreased itching and blister count, reduced urticarial plaques, and reduced eosinophilic inflammation (86, 87). Together, these clinical research and clinical trial data suggest that IgE autoantibodies in BP patients are involved in BP development likely through FcεRI-induced degranulation of MCs and basophils. However, pathogenic anti-BP180 IgE autoantibodies could also act on eosinophils in BP tissue injury since eosinophils express IgE receptors and are predominant infiltrating immune cells in BP (41).

## Animal Models of BP

**Figure 2:**
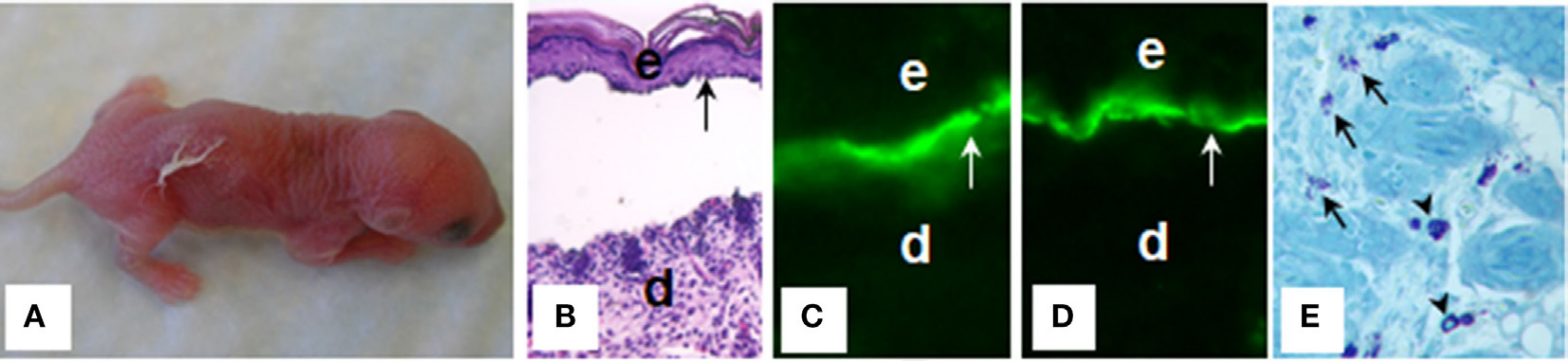
Mouse bullous pemphigoid. The anti-BP180 IgG induce extensive blistering disease in neonatal B6 mice clinically **(A)** and histologically **(B)**. The skin of these animals shows linear deposition of anti-BP180 IgG **(C)** and murine C3 **(D)** at the BMZ, as determined by direct IF. Toluidine blue staining shows resting and degranulating mast cells in the dermis **(E)**. d, dermis; e, epidermis; v, vesicle; arrow, the BMZ. Original magnification, 200× for panels **(B–D)**, 400× for panel **(E)**. **(E)** Arrows for degranulating mast cells, and arrow heads for normal resting mast cells.

Bullous pemphigoid autoantibodies were thought to be responsible for blister formation in BP; however, passive transfer of IgG autoantibodies from BP patients could not induce a BP-like disease in animals (88, 89). It turned out that BP autoantibodies reacting with NC16A domain that harbors immunodominant and potentially pathogenic epitopes fail to cross-react with mouse BP180; therefore, BP IgG autoantibodies cannot be tested for pathogenicity in a conventional passive transfer mouse model. In 1993, Liu et al. (90) subcloned a segment of the murine BP180 protein homologous with the human BP180 NC16A (mBP180 NC14A), generated rabbit polyclonal antibodies against mBP180 NC14A, and administrated the purified rabbit anti-mBP180 IgG intradermally or intraperitoneally into neonatal BALB/c mice. This experimental BP model reproduced all of the key clinical, histological, and immunopathological features of BP, including deposition of rabbit anti-mBP180 IgG and mouse complement C3 at dermal–epidermal junction, infiltration of inflammatory cells, and subepidermal blistering (90) (Figure 2). Anti-BP180 IgG-induced BP blistering required complement activation and neutrophil recruitment (91, 92). Subsequently, BP serum-purified IgG autoantibodies against BP180 or NC16A domain were also demonstrated to be pathogenic in BP180 humanized mouse models (93, 94).

BP180-specific IgE autoantibodies purified from serum of BP patients when passively transferred into human skin grafted onto athymic nude mice induced skin lesions that recapitulated the initial phase of disease. The features of the early phase of the disease are characterized by increased plaques and MC degranulation in comparison with injection of normal control human IgE (95). Lesional skin of the anti-BP180 IgE-injected mice also exhibited infiltration of neutrophils and eosinophils (95). However, it remains to be determined whether the pathogenic activity of anti-BP180 IgE depends on eosinophils, MCs, or both.

## Role of MCs in Experimental BP

To determine whether MCs were involved in experimental BP, Chen et al. (19) demonstrated that wild-type MC-sufficient mice administrated intradermally with pathogenic anti-mBP180 IgG developed BP disease with extensive MC degranulation in the upper dermis, which preceded infiltration of neutrophils and subsequent dermal–epidermal separation. In contrast, MC-deficient Kit*^W/W-v^* and Mgf*^Sl/Sl-d^* mice failed to develop BP (19). Moreover, these MC-deficient mice reconstituted with wild-type bone marrow-derived MCs, and polymorphonuclear leukocytes from these MC-deficient mice or by intradermal injection of IL-8 (a neutrophil chemoattractant) became susceptible to experimental BP (19). Blocking MC degranulation by treating MC-sufficient mice with an MC degranulation inhibitor also significantly reduced disease phenotype (19).

To determine the functional relationship between MCs and neutrophils, Chen et al. found that anti-BP180 antibody-induced neutrophil infiltration depends mainly on MCs in experimental BP (19). Without MCs, Kit*^W/W-v^* and Mgf*^Sl/Sl-d^* mice injected with pathogenic IgG show about 70% reduction of infiltrating neutrophils in the skin (96). Further examination of the experimental BP model also implicated macrophages in anti-BP180 IgG-triggered neutrophil infiltration in mice, and that macrophage-mediated neutrophil infiltration depends on MC activation (96). The findings that neutrophil recruitment is not completely impaired in MC-deficient mice in experimental BP suggest that at least two neutrophil recruitment pathways could exist: MC-dependent and MC-independent (96). Nevertheless, these data suggest a major role of MCs in infiltration of neutrophils into the dermis in this animal model setting.

Mast cells express surface receptors that directly bind the cleaved products of the activated complement cascade (97). Skin MCs express the C5aR (98), and upon the molecular interaction of C5a and C5aR, MCs degranulate, releasing several pro-inflammatory cytokines including TNF-α, IL-1, IL-6, and GM-CSF (99). Moreover, human C3a and C5a could degranulate MCs *in vitro* to release histamine and tryptase. Heimbach et al. (100) demonstrated that interaction of C5a–C5aR on MCs activated the p38 MAPK pathway that trigger MC degranulation and subsequent tissue injury and blister formation.

Mast cells store proteases in large quantities in the secretory granules, and these fully functional enzymes are a major class of inflammatory mediators (101, 102). Human cutaneous MCs contain a single chymase, and mouse MC protease-4 (mMCP-4) has been generally recognized as the likely homolog of the human chymase (103–105). Importantly, mMCP-4 can activate MMP-9, a key proteolytic enzyme for tissue injury in experimental BP (106). Interestingly, mMCP-4^−/−^ mice are resistant to anti-BP180 IgG-induced experimental BP (107). In experimental BP, mMCP-4 activates MMP-9 and directly cleaves BP180. mMCP-4, MMP-9, and other proteolytic enzymes work together to degrade BP180 and other hemidesmosomal proteins and proteins in extracellular matrix of the BMZ (107), leading to clinical and histological BP-like blistering.

**Figure 3:**
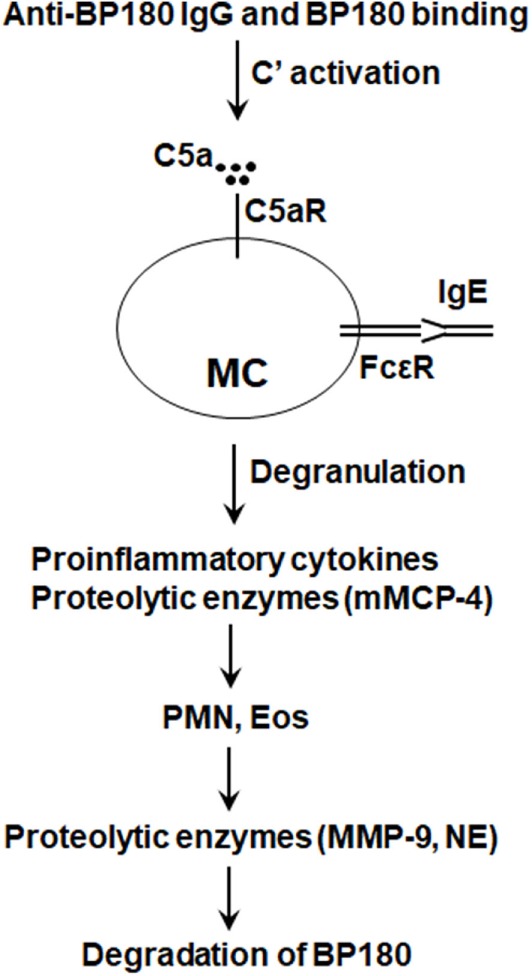
Proposed role of mast cells (MCs) in bullous pemphigoid (BP). Anti-BP180 IgG binding to BP180 on the surface of basal keratinocytes activates the complement (C), generating C5a. C5a acts on C5a receptor (C5aR) to cause MCs to degranulate and release pro-inflammatory cytokines/chemokines (e.g., TNFα) and proteolytic enzymes including mouse MC protease-4 (mMCP-4). Anti-BP180 IgE could also activate MCs. The released pro-inflammatory mediators interact with local cells to recruit neutrophils (PMN) and eosinophils (Eos). Infiltrating PMN and Eos, upon activation through interactions between immobilized anti-BP180 IgG/IgE and FcγR/FcεR, release neutrophil elastase (NE), MMP-9, and other proteolytic enzymes. mMCP-4 activates MMP-9 and also directly cleaves BP180 and other BP180-associated proteins in concert with MMP-9 and NE, resulting in subepidermal blistering.

Taken together, results of these studies using MC-deficient and C5aR and mMCP-4 knockout mice implicate a pathogenic role of MCs in BP (Figure 3). However, since the studies on the role of MCs in anti-BP180 IgG-induced experimental BP have been performed only in MC-deficient Kit*^W/W-v^* and Mgf*^Sl/Sl-d^* mice, KIT-independent MC-specific deletion mouse strains need to be tested to confirm or clarify the involvement of MCs in experimental BP.

## Role of MCs in Epidermal Bullosa Acquisita (EBA)

Epidermal bullosa acquisita is another autoimmune subepidermal blistering skin disease caused by autoantibodies against collagen VII (108). Experimental EBA can be induced by passive transfer of anticollagen VII IgG (109, 110). Immunopathogenically, experimental EBA shares many key features with experimental BP such as their dependency on complement, C5a-C5aR signaling, and neutrophils (109, 111). However, anticollagen VII IgG causes similar disease severity in both wild-type control and MC-deficient Kit*^W/W-v^* mice (112). KIT-independent MC-specific deletion mice are also not protected from experimental EBA (112). These studies demonstrate that MCs do not contribute to experimental EBA, further emphasizing a need to revisit the role of MCs in experimental BP using KIT-independent MC-specific deletion strains.

## Concluding Remarks

We presented several lines of BP animal model evidence, together with clinical observations, implicating that MCs are likely to be involved in the immunopathogenesis of BP. The role of MCs in experimental BP, however, was investigated exclusively in KIT-dependent MC-deficient mice. Based on the observed discrepancies in different MC-deficient models of EAE, RA, and EBA, it is necessary to perform anti-BP180 IgG-induced BP studies in KIT-independent MC-specific deletion strains to clarify whether MCs play a role in BP.

Bullous pemphigoid patients also have anti-BP180 IgE autoantibodies, which are involved in tissue injury (95); therefore, a potential role of MCs in anti-BP180 IgE-induced BP should be determined in both KIT-dependent and KIT-independent MC-deficient strains. Future studies could also investigate whether and how MCs interact with anti-BP180 IgG, anti-BP180 IgE, and eosinophils during disease development. Giving the fact that MCs have a variety of immunomodulatory activities (14), MC contribution to BP could be multifaceted. Advanced tools need to be developed to clarify and fully appreciate the contribution of MCs to BP and help uncover new therapeutic targets for this potentially fatal skin autoimmune disease.

## Author Contributions

HF, YZ, NL, GW, and ZL wrote the manuscript; HF, YZ, and NL prepared the figures.

## Conflict of Interest Statement

The authors declare that the research was conducted in the absence of any commercial or financial relationships that could be construed as a potential conflict of interest.
